# Endolymphatic hydrops and intracranial hypertension: a quantitative MRI analysis

**DOI:** 10.3389/fneur.2025.1751689

**Published:** 2025-12-18

**Authors:** Anne R. J. Péporté, Joana Kostova, Jatta Berberat, Gustav Andreisek, Fabian Schön, Franca Wagner

**Affiliations:** 1 Department of Radiology, Cantonal Hospital Frauenfeld, Frauenfeld, Switzerland; 2 Department of Diagnostic and Interventional Neuroradiology, Cantonal Hospital Aarau, Aarau, Switzerland; 3 Department of Radiology, Cantonal Hospital Münsterlingen, Münsterlingen, Switzerland; 4 Institute of Diagnostic and Interventional Radiology, University Hospital Zurich, Zurich, Switzerland; 5 Department of Otorhinolaryngology and Head and Neck Surgery, University Hospital Bern, Bern, Switzerland

**Keywords:** endolymphatic hydrops, idiopathic intracranial hypertension, Meckel’s cave, Menière’s disease, MRI, MRI biomarkers, transverse sinus stenosis, vestibulocochlear disorders

## Abstract

**Objective:**

This study investigated the prevalence and association of magnetic resonance imaging (MRI) imaging markers indicative of idiopathic intracranial hypertension (IIH) in patients diagnosed with endolymphatic hydrops (EH). The objective was to elucidate potential pathophysiological links between inner ear fluid dysregulation and alterations in intracranial pressure.

**Methods:**

A total of 108 adult patients with dedicated MRI and delayed post-contrast (hydrops) sequences obtained for assessment of auditory/vestibular symptoms between 01/2020 and 06/2025 were retrospectively reviewed. EH grading, nerve volumes of the cochlear nerve, common vestibular trunk and facial nerve, IIH imaging features (e.g., Meckel’s cave dilatation, optic nerve findings, venous sinus stenosis), and clinical symptoms were recorded. The prevalence and co-occurrence of EH and IIH features were statistically analyzed.

**Results:**

Vestibular EH (grade 2) was noted in 71.3% (right) and 60.2% (left) of patients and cochlear EH (grade 2) in 42.6% (left) and 34.3% (right) of patients. IIH-related imaging markers were common: bilateral Meckel’s cave dilatation (60.2%), partially empty sella turcica (50.9%), bilateral optic nerve sheath dilation (57.4%), optic nerve head enhancement on delayed FLAIR sequences (67.6%), and intrinsic bilateral transverse sinus stenosis (26.9%). Statistically significant associations were identified between vestibular EH severity and optic nerve sheath dilation (*p* = 0.0368), optic nerve tortuosity (*p* = 0.0309), slit-like lateral ventricles (*p* = 0.0023), and increased subcutaneous fat thickness in the scalp and neck (*p* = 0.003). Conversely, intrinsic bilateral transverse sinus stenosis was negatively correlated with EH severity (*ρ* = −0.228, *p* = 0.017). Overlap analyses demonstrated that many patients with moderate to severe EH exhibited multiple IIH imaging features concomitantly.

**Conclusion:**

MRI findings demonstrate a frequent coexistence of EH and radiological biomarkers of IIH. This observation supports a potential pathophysiological association between inner ear fluid dysregulation and elevated intracranial pressure, underscoring the need for prospective studies integrating clinical outcomes with advanced MRI-based assessments of fluid dynamics.

## 1 Introduction

Endolymphatic hydrops (EH) is characterized by pathological distension of the membranous labyrinth due to excessive endolymph—the potassium-rich fluid within the membranous labyrinth. EH serves as a key radiological biomarker of both primary [Menière’s disease (MD)] and secondary hydropic ear diseases. Patients with EH typically present with recurrent episodes of vertigo, fluctuating sensorineural hearing loss, tinnitus, and a sensation of aural fullness (1, 2). The precise anatomical and neurophysiological mechanisms underlying these symptoms remain poorly defined.

Advances in magnetic resonance imaging (MRI), particularly high-resolution 3 T scans, have enabled detailed *in vivo* visualization and morphometric evaluation of the vestibulocochlear nerve complex. Historically, EH diagnosis was based on clinical presentation and audiometric testing, as direct imaging of EH was not feasible. However, over the past decade, advances in MRI—particularly dedicated delayed post-gadolinium techniques—have enabled *in vivo* visualization of the endolymphatic and perilymphatic compartments. This allows direct assessment and semi-quantitative grading of EH and identification of imaging biomarkers. Such biomarkers include the vestibular EH ratio, cochlear and vestibular hydrops grading, vestibular herniation into the ampulla of the semicircular canals, and patterns of perilymphatic enhancement relevant to MD (3–5). The detection of EH with MRI has been incorporated as a diagnostic criterion in the latest *guidelines on Ménière’s disease* issued by the Japan Society for Equilibrium Research (6).

Whereas EH is a well-established histopathological correlate for MD, recent reports highlight its presence in broader neurological contexts. These include patients with intracranial pressure (ICP) abnormalities, such as idiopathic intracranial hypertension (IIH) and spontaneous intracranial hypotension (SIH). IIH is a disease characterized by elevated ICP and is often associated with conditions such as transverse sinus stenosis (TSS) and impaired cerebrospinal fluid (CSF) dynamics, rather than being idiopathic in the strictest sense. IIH typically affects obese, middle-aged women who classically present with headache, tinnitus, and visual changes (7–9). Evaluation includes lumbar puncture with measurement of opening pressure (values ≥25 cm H_2_O are considered diagnostic in the appropriate clinical context) alongside intracranial imaging to rule out other causes of raised ICP. There are many overlaps in the clinical presentations of EH and IIH, including a high prevalence of vertigo, tinnitus, aural fullness, sensorineural hearing loss, and headache in both groups of patients (10–13).

Several studies underline that patients with raised ICP may exhibit audiovestibular symptoms typical of EH. These include vertigo, tinnitus, and hearing loss, which are linked to inner ear fluid dysregulation secondary to altered CSF pressure dynamics. Redon et al. (14) further suggested that neurological disorders with CSF pressure fluctuations can result in secondary forms of EH, expanding the traditional etiological framework beyond primary EH/MD. The relationship between cochlear and vestibular pathology in inner ear disorders is complex, with evidence suggesting that the cochlear and vestibular end-organs may be differentially susceptible to underlying fluid dysregulation or influenced by additional systemic factors such as ICP dynamics (15). This study investigates this complex interplay through a novel pressure-dynamics imaging approach using quantitative MRI biomarkers.

To the best of our knowledge, no clinical studies have so far investigated the prevalence of surrogate MRI markers of IIH among patients with EH. As lumbar puncture is not routinely performed in the workup for EH, radiographic signs of IIH were identified as surrogate indicators for the purpose of this study. MRI markers were prioritized over lumbar puncture in this population due to the retrospective design and focus on non-invasive imaging in audiovestibular symptom workup, as lumbar puncture is not routine for EH assessment. This approach allows the evaluation of ICP-related changes using established MRI biomarkers without subjecting patients to invasive procedures.

## 2 Methods

### 2.1 Patient population

The study was approved by the Ethics Committee of Eastern Switzerland (Req-2024-01599 EKOS 24/236). The requirement for informed consent was waived. A retrospective review of our institutional digital database was carried out for delayed contrast-enhanced MRI scans of the inner ear for hydrops imaging between 01/2020 and 06/2025.

Patients with local and systemic diseases directly involving the temporal bone were excluded. Further exclusion criteria were severe motion artifacts and incomplete imaging. Inclusion criteria were age >18 years and available high-resolution 3 T MRI scans encompassing the internal auditory canal (IAC) and including delayed post-gadolinium MRI sequences. The initial retrospective review of our institutional digital database for MRI of the inner ear revealed 132 potential participants with 144 MRI scans of the neck.

MRI scans were excluded for the following reasons: poor imaging quality due to severe image distortion (*n* = 7), severe motion artifacts (*n* = 11), incomplete imaging (*n* = 6), and local/systemic diseases directly involving the temporal bone. From an initial retrospective review of 132 potential participants yielding 144 MRI scans, 108 patients met inclusion criteria (age >18 years with high-resolution 3 T MRI including delayed post-contrast sequences for inner ear assessment).

### 2.2 MRI acquisition

All participants underwent 3 Tesla MRI scanning (Siemens Magnetom Vida 3 Tesla, Philips Achieva 3 Tesla) using standardized protocols optimized to visualize inner ear structures, the vestibulocochlear nerve complex, and the endo- and perilymphatic spaces. Acquisitions included 3D high-resolution T2-weighted sequences and delayed post-gadolinium sequences (3D FLAIR and 3D T2w inversion recovery sequence 4 h post-contrast) (Table 1).

**Table 1:** Example of detailed scanning parameters of the specific sequences of our inner ear endolymphatic hydrops MRI protocol.^a^

Sequence	Plane	Field of view (mm)	Echo time (ms)	Repetition time (ms)	Flip angle (°)	Slice thickness (mm)
T2	3D tra	150 × 150	Shortest	1,500	90	0.8
FLAIR (4 h post-contrast)	3D tra	250 × 250	340	4,800	40	0.5–0.7
T2 inversion recovery (4 h post-contrast)	3D tra	75 × 150	177	6,000	180	1

Tra, transverse; cor, coronal; mm, millimeters; ms, milliseconds.

a 3 Tesla MRI Scanner (Philipps Magnetom Achieva).

### 2.3 MRI assessment and volumetry

#### 2.3.1 Volumetric measurement

**Figure 1:**
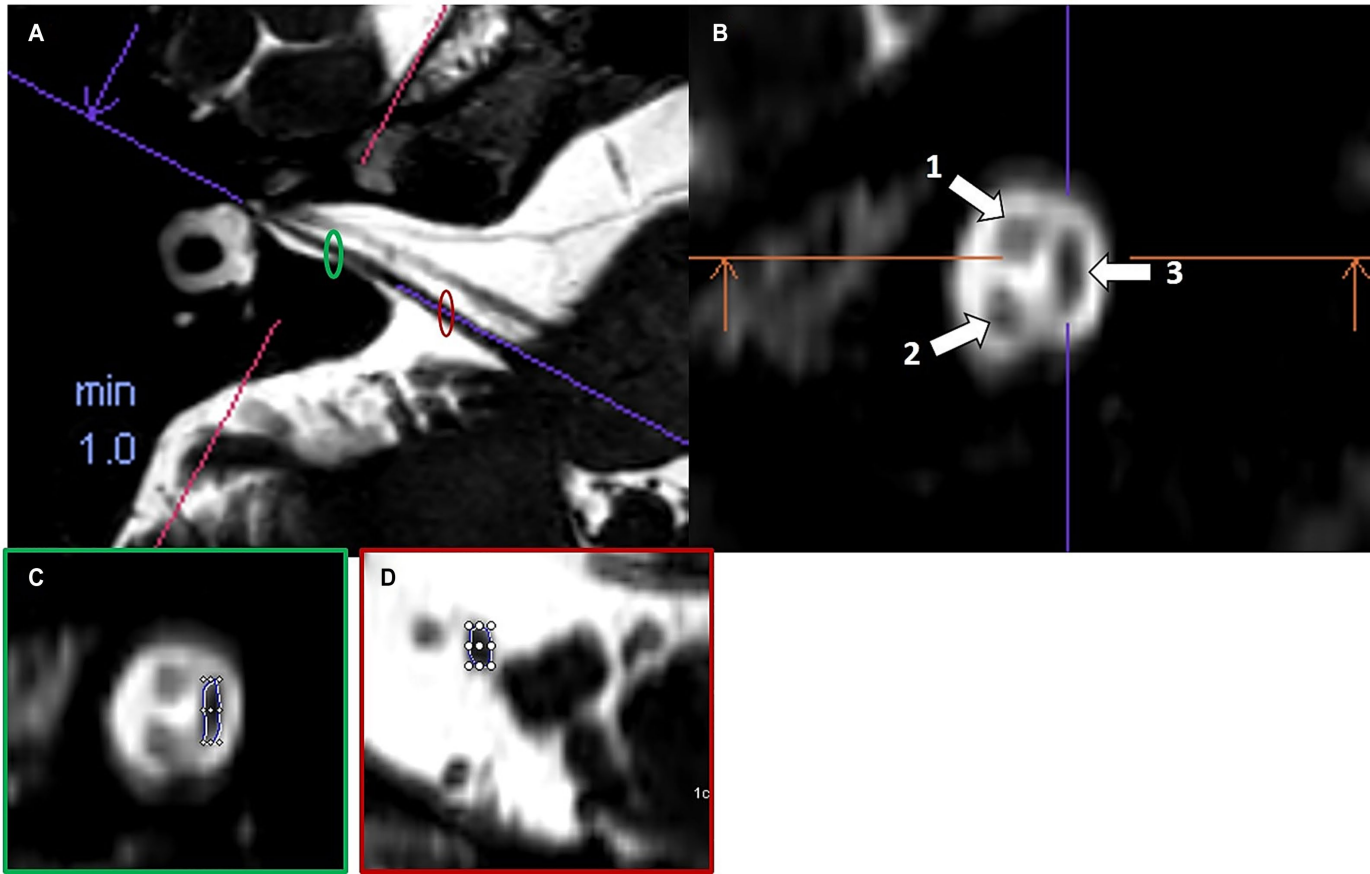
Example of volumetric measurement of the common vestibular nerve. Axial T2w SPACE image **(A)** at the level of the internal auditory canal showing the orientation of the common vestibular nerve and location of the oblique sagittal 0.8 mm multiplanar reformat from which neural volumetric measurements were obtained. The corresponding oblique sagittal reformatted image **(B)** at the fundus of the internal auditory canal is positioned lateral to the cochlear aperture with the facial nerve (1), cochlear nerve (2) and the common vestibular nerve (3). Panels **(C,D)** show sections of the volumetric drawing of the common vestibular nerve, one example in the intrameatal segment (green ellipse) and one example in the cisternal segment (red ellipse).

Cochlear and vestibular nerve volumes were measured by AP (a board-certified neuroradiologist holding the European Diploma in Head and Neck Radiology, with more than 6 years’ experience in head and neck imaging), who was blinded to clinical data. A semi-automated segmentation software package (Syngo.via, Siemens Healthineer, version VB80D) was used for volumetric measurements. Bilateral cochlear nerve and vestibular nerve complex volumes were obtained by axis-corrected measurement from the cerebellopontine angle to the IAC fundus. Volume of interest regions were inserted throughout the course of the cranial nerves. The cumulative area of each MRI slice was then calculated by the software to obtain the entire nerve volume. The partial blurring seen at the margins of the reconstructed nerves (penumbra effect) was managed by delineating contours at the midpoint between the central low-signal area and the surrounding high-signal region (Figure 1).

The volume of the common vestibular nerve was measured, rather than measuring the superior and inferior vestibular nerve volumes separately. These two branches are often not completely anatomically separated throughout their course through the IAC. Anatomical studies have shown that the vestibular nerve divides into its superior and inferior branches only near the lateral end of the canal, typically at or just beyond the falciform crest. For most parts of their intrameatal course, these divisions cannot be distinctly visualized or separated, either by cadaveric dissection or by imaging, because they are commonly fused. In many specimens, the superior and inferior vestibular nerves appear as a single structure (the common vestibular nerve), and only become clearly distinguishable at the fundus of the IAC. Because of this anatomical continuity, volumetric measurements of the common vestibular trunk are most reliable, as attempting to distinguish and separately measure the individual branches would not be consistently feasible or reproducible and do not accurately reflect the try anatomy of the nerves (16, 17).

#### 2.3.2 EH grading

Two readers (AP and FS, a general radiologist with 10 years’ experience in general radiology) independently graded cochlear and vestibular EH, based on established MRI criteria (18). Ears were categorized into one of four distinct grades (no EH, grade 1, grade 2, or grade 3), as described by Bernaerts et al. (18). Both readers were blinded to clinical data. Control ears were those ears without any features of EH on MRI according to any EH grading system.

#### 2.3.3 MR evaluation of IIH findings

MRI scans were evaluated for IIH biomarkers by AP, who was blinded to clinical data. The MRI IIH biomarkers evaluated were as follows.

##### 2.3.3.1 Arachnoid outpouchings

###### 2.3.3.1.1 Empty/partial sella sign (ES/PS)

**Figure 2:**
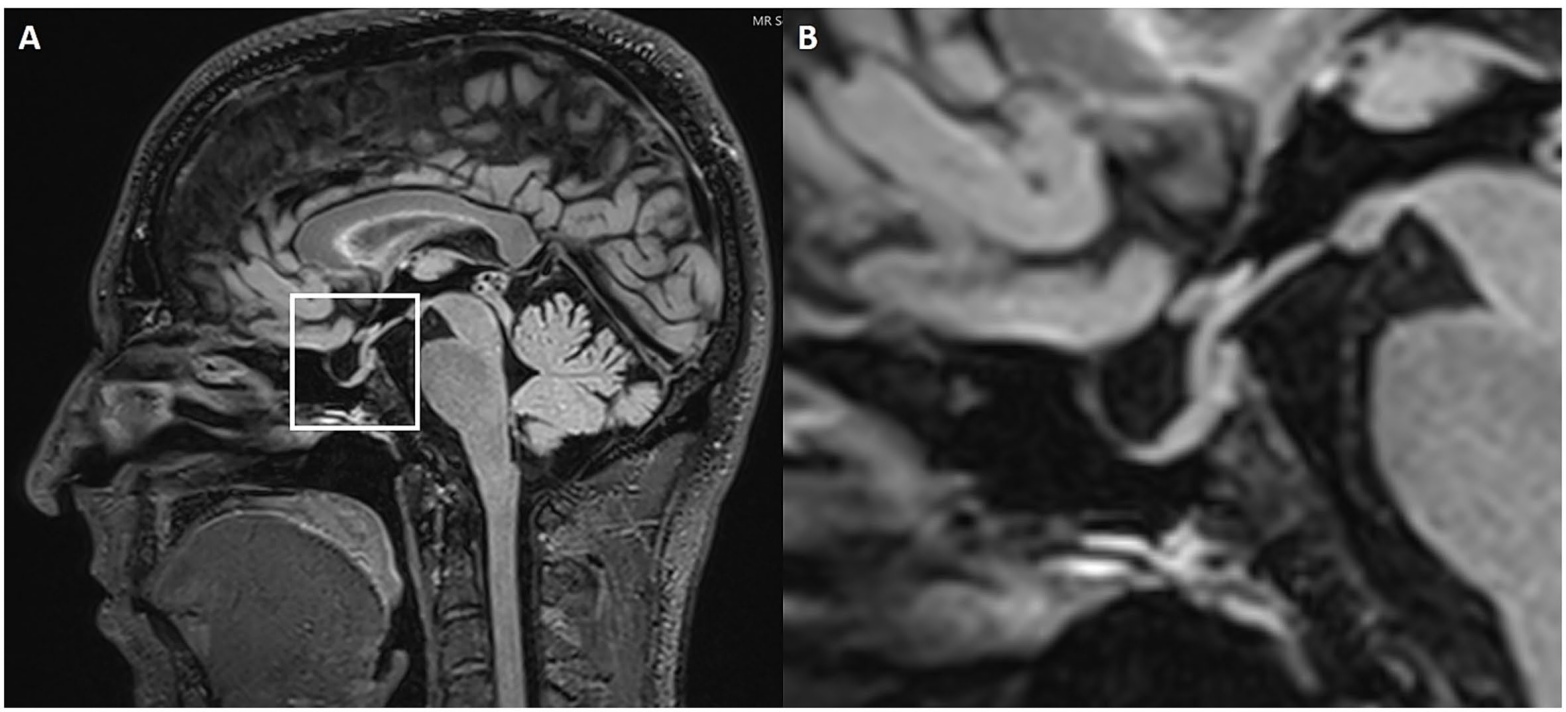
Partially empty sella. Sagittal 3D FLAIR **(A)** MRI with magnification **(B)**. The white box highlights the sella turcica and pituitary gland. The magnified image shows a slight enlargement of the pituitary fossa, which is filled with CSF.

ES/PS was characterized according to established criteria and conventions documented in the published literature (19–24). This included the application of age- and sex-adjusted reference standards for ES/PS, as well as validated cutoff criteria for empty or partial sella signs linked to IIH. Pituitary height was measured in millimeters at the widest visible part of the gland parenchyma on either a midsagittal pre-contrast T1- or T2-weighted image (Figure 2). A pituitary height less than 4.8 mm was used as the diagnostic threshold for ES/PS. The data were categorized and tabulated based on whether ES/PS was present or absent.

###### 2.3.3.1.2 Dilated Meckel’s cave

**Figure 3:**
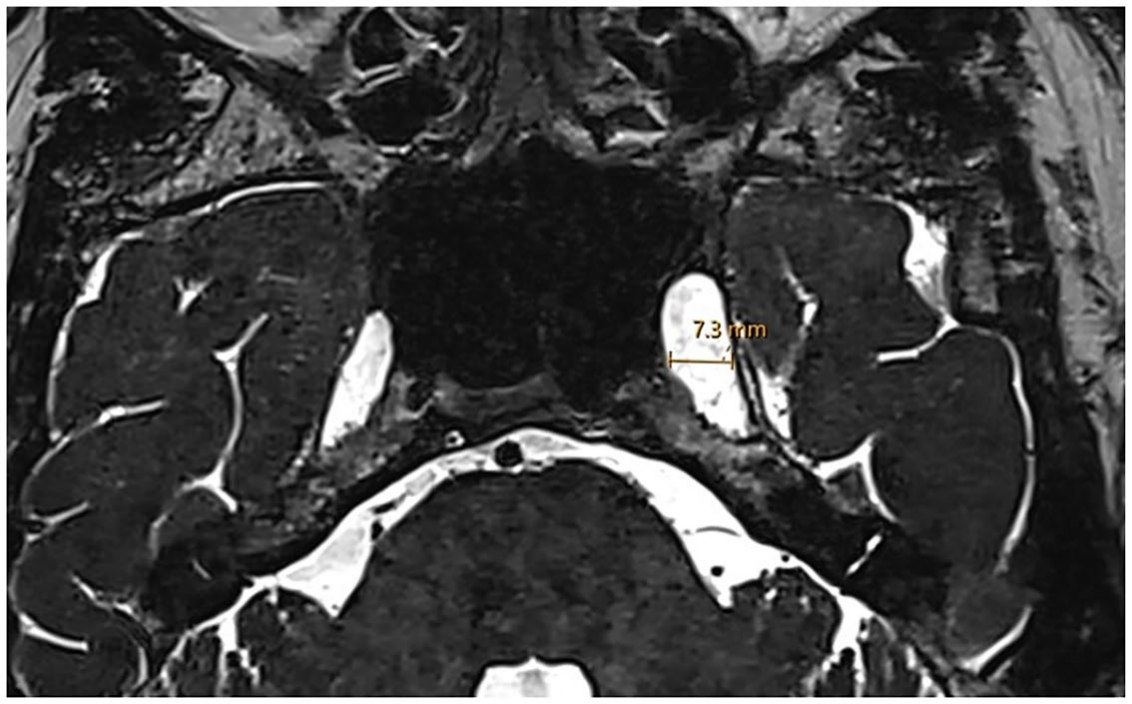
Enlarged Meckel’s cave. Axial 3D T2w SPACE MRI shows an enlarged left Meckel’s cave with a transverse diameter of 7.3 mm.

Assessment of Meckel’s cave dilatation was performed on 3D high-resolution T2-weighted axial and coronal sequences. Axial T2-weighted images were reviewed for evidence of a prominent or increased fluid signal expanding the Meckel’s cave without distorting the contours (considered prominent Meckel’s cave), or a frank meningocele. A meningocele was defined as bulging of the dura mater, arachnoid and CSF into Meckel’s cave (25, 26). In doubtful cases, the transverse diameter of Meckel’s cave was measured at its largest point (Figure 3). A diameter greater than 5 mm was defined as dilated based on established radiological criteria (27).

###### 2.3.3.1.3 Arachnoid pits/small meningoceles

Arachnoid granulations in the temporal bone and sphenoid region were assessed on high-resolution T2-weighted and contrast-enhanced T1-weighted MRI sequences. These lesions characteristically appear isointense to CSF on T1- and T2-weighted images, show no contrast enhancement, and lack solid mass components. Meningoceles were identified as herniations of CSF-filled sac-like protrusions through bony defects often associated with arachnoid granulations. They were visible as CSF-isointense outpouchings on T2-weighted MRI sequences, extending beyond the normal anatomical boundaries (25).

##### 2.3.3.2 Optic nerve and optic nerve sheath alterations

###### 2.3.3.2.1 ONS tortuosity/dilation

**Figure 4:**
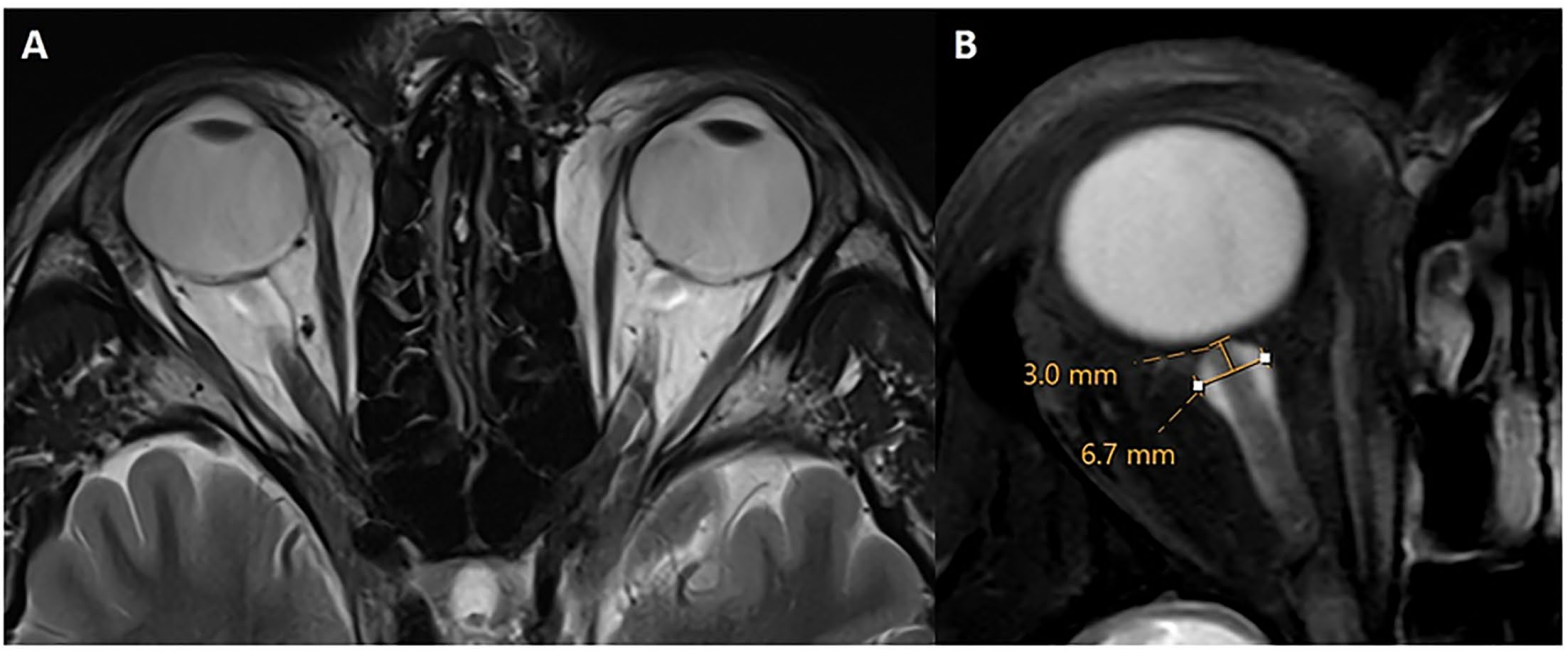
Optic nerve sheath dilation and tortuosity. Axial T2w without **(A)** and with fat suppression **(B)** through the level of the optic nerve. There is bilateral tortuosity of the optic nerve and its sheath on both sides with a diameter of 6.7 mm in this case. Measurements of optic nerve sheath diameter were obtained 3 mm posterior to the optic globe.

The intraorbital optic nerve (ON) and its arachnoid sheath were evaluated and measured on an axial 3D T2-weighted image with or without fat suppression, following established conventions (19, 28). ON tortuosity was defined as deviation of the intraorbital segment of the ON medially or laterally to an extent greater than or equal to the nerve width. Alternatively, failure to visualize a central segment of the ON, while the nerve is clearly seen both proximal and distal to that segment within the same imaging plane, was considered diagnostic of IIH. Optic nerve sheath (ONS) dilation was also assessed following established criteria (19, 28–35). Measurements were acquired at a consistent location along the distal intraorbital segment, 3 mm behind the posterior aspect of the globe (Figure 4). At this site, the ONS diameter was recorded, with a diameter of ≥5.60 mm considered indicative of IIH. While there is no single definitive threshold, values of >5.60 mm have been proposed (36). ONS tortuosity and dilation were combined into a single categorical variable (present/absent), reflecting their presence either independently or together.

###### 2.3.3.2.2 ON head enhancement

ON head enhancement was evaluated on 3D T1-weighted post-gadolinium images and on delayed 4-h post-contrast FLAIR images, which offer increased sensitivity to subtle enhancement. Abnormal enhancement at the ON head reflects disruption of the blood–nerve barrier due to elevated ICP and correlates with papilledema or ONS distension in IIH (37). The differential diagnosis of anterior ischemic optic neuropathy was excluded by careful review of diffusion-weighted imaging to rule out restricted diffusion. Additionally, ON or ONS space-occupying lesions were excluded by ruling out mass-like enhancement. Enhancement was recorded as present or absent bilaterally.

##### 2.3.3.3 Venous outflow

###### 2.3.3.3.1 Presence of TSS (extrinsic/intrinsic)

**Figure 5:**
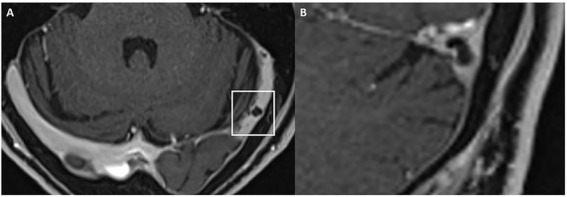
Left transverse sinus stenosis. Axial **(A)** and coronal **(B)** 3D T1w post-contrast MRI showing an arachnoid granulation in the left transverse sinus consistent with an intrinsic stenosis.

Bilateral TSS is associated with IIH in 90% of patients (38). TSS was assessed on contrast-enhanced MR venography or 3D high-resolution post-gadolinium T1-weighted sequences by identifying focal filling defects or caliber narrowing, typically caused by arachnoid granulations, septations or intrinsic/extrinsic compression (Figure 5). Both extrinsic and intrinsic stenoses were recorded, as they are key imaging hallmarks of IIH (39, 40).

##### 2.3.3.4 Ancillary findings

###### 2.3.3.4.1 Changes in ventricular size

A slit-like appearance of the lateral ventricles due to their narrowing and collapse is a sign of IIH.

**Figure 6:**
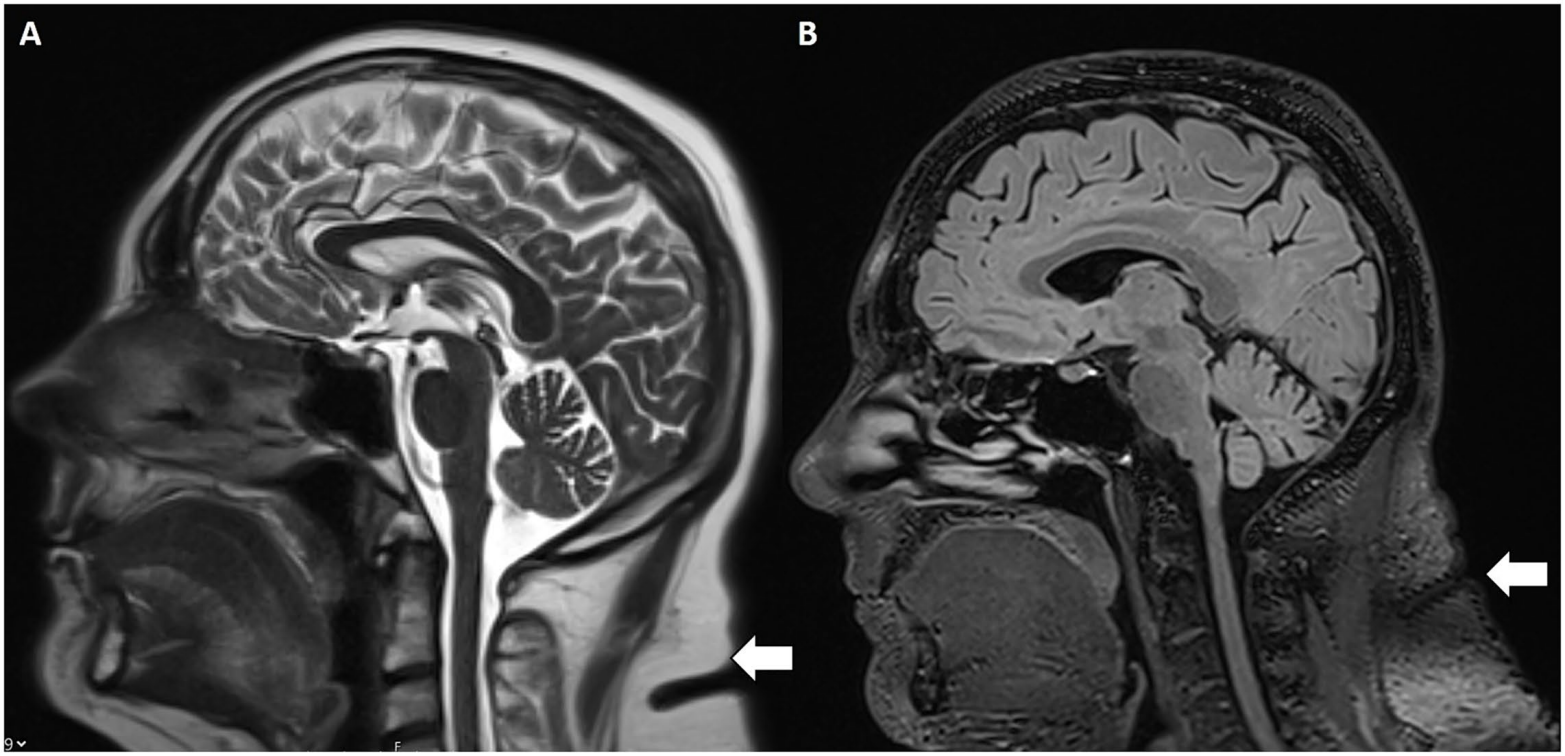
Increased subcutaneous nuchal fat. Sagittal T2w **(A)** and FLAIR **(B)** MRI with increased subcutaneous fat (white arrow) at the C2–C3 intervertebral disc space.

Increased subcutaneous fat thickness in the scalp and neck were evaluated as further IIH MR findings (22). Specifically, patients with IIH demonstrated significantly greater scalp fat thickness at the level of the coronal suture and increased neck fat thickness at the C2–C3 vertebral body interspace (Figure 6) compared to controls with incidental empty sella turcica. These findings support subcutaneous fat thickness as a potential imaging marker associated with intracranial hypertension.

### 2.4 Clinical data and symptom assessment

Data on clinical symptoms collected from patient records included presence or absence of hearing loss, vertigo, tinnitus, sudden hearing loss and aural fullness, recorded for each side.

### 2.5 Statistical analysis

Statistical analyses were performed by JK and JB. For the univariable analyses of associations between IIH-related imaging signs and EH outcomes, chi-square tests and Fisher’s exact tests were applied to binary variables, and Spearman’s correlation was used for ordinal variables. *p*-values were adjusted for multiple comparisons within each outcome using the false discovery rate method. Multivariable logistic regression models for EH outcomes were adjusted for age and sex, with model selection guided by the strongest univariable associations and data availability. Adjusted odds ratios with 95% confidence intervals are reported for analyzed biomarkers.

All analyses were performed using Python Version: 3.11.6; statsmodels 0.14.0; pandas 2.2.3; and scikit-learn 1.6.1.

## 3 Results

### 3.1 Patient population

A study cohort of 108 patients diagnosed with EH met the inclusion criteria. Mean age was 53.6 ± 15.8 years and there was a slight female predominance (61/108; 56.5%).

### 3.2 MRI assessment and volumetry

#### 3.2.1 Volumetric measurement

Cranial nerve volumetry showed average cochlear nerve volumes of 9.52 ± 4.92 mm^3^ on the left side and 9.10 + 5.90 mm^3^ on the right. Similarly, volumes of the vestibular and facial nerves showed no significant asymmetries at the cohort level.

Evaluation revealed a mean left cochlear nerve volume of 9.52 ± 4.92 mm^3^ and a mean right cochlear nerve volume of 9.10 ± 5.9 mm^3^. The common vestibular nerve trunk measured 19.78 ± 10.18 mm^3^ on the right and 18.95 ± 9.7 mm^3^ on the left, while facial nerve volumes averaged 13.71 ± 5.83 mm^3^ (right) and 12.35 ± 5.45 mm^3^ (left).

No statistically significant differences in cochlear nerve volume were found between cochlear EH grades 1–3 (*p* = 0.057), despite a nonsignificant tendency toward larger volumes in patients with mild EH grades. There was no significant difference in vestibular nerve volume between vestibular EH grades 1 and 2 (*p* = 0.64). Comparisons of cochlear nerve volume between affected and unaffected ears in patients with unilateral hearing loss also showed no significant differences (*p* = 0.56).

#### 3.2.2 EH grading

In the vestibular system, grade 2 EH was observed in 71.3% (77/108) of right and 60.2% (65/108) of left vestibules, while grade 1 EH was seen in 15.7% (17/108) on the right and 21.3% (23/108) on the left. Only one participant (0.9%) had grade 3 EH on the left side, and none had grade 3 EH on the right side.

Similarly, in the cochlea, grade 2 EH predominated, affecting the left side in 42.6% (46/108) and the right side in 34.3% (37/108) of patients. Grade 1 EH was present in 18.5% (20/108) of left and 19.4% (21/108) of right cochleae, while grade 3 EH was rare, observed in only one right cochlea (0.9%) and none on the left.

Concurrent vestibular and cochlear EH was highly prevalent, with 71/108 patients (65.7%) exhibiting EH in BOTH compartments regardless of severity grade. Overall, these findings indicate that moderate (grade 2) EH was the most prevalent pattern, with severe (grade 3) involvement being uncommon.

### 3.3 MRI evaluation of IIH findings

MRI indicators suggestive of IIH were prevalent in the cohort.

#### 3.3.1 Arachnoid outpouchings

Although partially empty sella turcica was present in 50.9% (55/108) and the prevalence of bilateral Meckel’s cave dilatation (transverse diameter >5 mm) was high (60.2%; 65/108) among EH patients, Spearman’s test did not reveal a significant correlation with EH severity. Arachnoid granulations or meningoceles were uncommon and no statistical correlation was noted.

#### 3.3.2 ON and ONS alterations

Bilateral dilation of the ONS was found in two-thirds (57.4%; 62/108) of patients and bilateral ON tortuosity in the vertical or horizontal planes in 49.1% (53/108).

Bilateral ON head enhancement had a high prevalence, especially on delayed FLAIR sequences (67.6%; 73/108).

A Spearman correlation analysis demonstrated a weak but statistically significant positive correlation between the presence of ON tortuosity and the severity of bilateral vestibular EH (*p* = 0.0309).

Fisher’s exact test revealed a statistically significant association of bilateral dilated ONS with vestibular EH (*p* = 0.0368).

#### 3.3.3 Venous outflow

Bilateral intrinsic TSS was present in one-third of the patients (26.9%; 29/108), while unilateral intrinsic TSS was less common (15/108; 13.9% on the left and 9/108; 8.3% on the right). Unilateral or bilateral extrinsic TSS was infrequent.

Univariable analyses revealed significant negative associations between venous sinus abnormalities and EH. Spearman correlation demonstrated a significant negative relationship between intrinsic bilateral TSS and EH severity (*ρ* = −0.228, *p* = 0.017). Additionally, chi-square and Fisher’s exact tests showed that intrinsic bilateral TSS was associated with decreased odds of bilateral EH (OR = 0.262, *p* = 0.013).

#### 3.3.4 Ancillary findings

Slit-like lateral ventricles were present in 38.9% (42/108) of patients. A chi-square analysis identified a strong positive and statistically significant association between the presence of slit-like ventricles and vestibular EH (*χ*^2^ = 15.375, *p* = 0.0023).

The prevalence of increased subcutaneous fat thickness of the scalp and neck was 42.6% (46/108). Multivariate proportional odds regression analysis demonstrated a significant association between increased subcutaneous fat thickness and cochlear EH (*p* = 0.003).

Clinically, vertigo was reported in 69.4% (75/108) of cases, sensorineural hearing loss in 70.4% (76/108), and tinnitus in 44.4% (48/108). Sudden hearing loss was documented in 21.3% (23/108).

Overlap analysis revealed that many individuals with moderate to severe EH also exhibited at least two radiological features consistent with IIH.

A summary of the results is presented in Table 2.

**Table 2:** Descriptive statistics.

Category	Variable	Mean (SD)	*N* (%)
Demographics	Age (years)	53.6 (15.8)	
	Sex (female)		61 (56.5)
Imaging: CN volumetry	Left cochlear nerve volume (mm^3^)	9.52 (4.92)	
	Right cochlear nerve volume (mm^3^)	9.10 (5.90)	
	Left vestibular nerve volume (mm^3^)	18.95 (9.7)	
	Right vestibular nerve volume (mm^3^)	19.78 (10.18)	
	Left facial nerve volume (mm^3^)	12.35 (5.45)	
	Right facial nerve volume (mm^3^)	13.71 (5.83)	
MRI: EH grading	Vestibular EH grade 1, left		23 (21.3)
	Vestibular EH grade 1, right		17 (15.7)
	Cochlear EH grade 1, left		20 (18.5)
	Cochlear EH grade 1, right		21 (19.4)
	Vestibular EH grade 2, left		65 (60.2)
	Vestibular EH grade 2, right		31 (28.7)
	Cochlear EH grade 2, left		46 (42.6)
	Cochlear EH grade 2, right		37 (34.3)
	Vestibular EH grade 3, left		1 (0.9)
	Vestibular EH grade 3, right		0 (0)
	Cochlear EH grade 3, left		0 (0.0)
	Cochlear EH grade 3, right		1 (0.9)
IIH imaging markers	Meckel’s cave dilatation		65 (60.2)
	Empty/partial empty sella		55 (50.9)
	Bilateral ONS dilatation		62 (57.4)
	Bilateral ON tortuosity		53 (49.1)
	Bilateral ON head enhancement		73 (67.6)
	Intrinsic bilateral TSS		29 (26.9)
	Slit-like ventricles		42 (38.9)
	Increased scalp/neck subcutaneous fat		46 (42.6)
Clinical symptoms	Vertigo		75 (69.4)
	Sensorineural hearing loss		76 (70.4)
	Tinnitus		48 (44.4)
	Sudden hearing loss		23 (21.3)

Summary of demographic characteristics, quantitative imaging findings, and prevalence of clinical and radiological markers in 108 patients with endolymphatic hydrops. Data are presented as mean (standard deviation) for continuous variables and number (percentage) for categorical variables.

SD, standard deviation; CN, cranial nerve; EH endolymphatic hydrops; IIH, idiopathic intracranial hypertension; ONS optic nerve sheath; ON, optic nerve.

### 3.4 Association between EH severity and IIH MRI biomarkers

To compare IIH-related MRI biomarkers across EH severity, patients were stratified into a no EH reference group, an any EH group, and a grade 2 vestibular EH subgroup. The prevalence of key IIH markers, including partially empty sella, bilateral ONS dilation, bilateral Meckel’s cave dilation, slit-like ventricles, and bilateral ON head enhancement, was consistently higher in EH-positive groups than in the no EH group, with several associations reaching statistical significance (Table 3). Intrinsic bilateral TSS showed an opposite pattern, with higher prevalence in the no EH group, supporting a negative association with EH severity (Table 3).

**Table 3:** Association between endolymphatic hydrops (EH) severity and IIH MRI biomarkers.

IIH biomarker	No EH (*n* = 13)	Any EH (*n* = 95)	Grade 2 EH (*n* = 77)	*χ*^2^/Fisher *p*-value
Partially empty sella	2 (15.4%)	49 (51.6%)	42 (54.5%)	***p* = 0.032**
Bilateral ONS dilation	1 (7.7%)	41 (43.2%)	37 (48.1%)	***p* = 0.012**
Bilateral Meckel’s cave dilation	3 (23.1%)	52 (54.7%)	46 (59.7%)	***p* = 0.045**
Slit-like ventricles	0 (0%)	24 (25.3%)	21 (27.3%)	***p* = 0.018**
Bilateral ON head enhancement (T1 + Gd)	2 (15.4%)	55 (57.9%)	49 (63.6%)	***p* = 0.008**
Intrinsic bilateral TSS	4 (30.8%)	18 (18.9%)	12 (15.6%)	***p* = 0.041** (negative assoc.)

Chi-square/Fisher exact tests comparing IIH biomarker prevalence across EH severity groups (no EH *n* = 13; any EH *n* = 95; grade 2 vestibular EH *n* = 77). Significant associations (*p* < 0.05) shown in bold. “No EH” group serves as baseline comparator. TSS, transverse sinus stenosis; ONS, optic nerve sheath; ON, optic nerve. Patients with grade 2 vestibular EH (*n* = 77) commonly had ≥2 IIH markers combined (e.g., empty sella + ONS dilation + Meckel’s cave), while no-EH patients (*n* = 13) rarely did (*χ*^2^ = 12.4, *p* = 0.002).

Patients with grade 2 vestibular EH (*n* = 77) commonly had ≥2 IIH markers combined (e.g., empty sella + ONS dilation + Meckel’s cave), while no-EH patients (*n* = 13) rarely did (*χ*^2^ = 12.4, *p* = 0.002).

## 4 Discussion

This study demonstrates a striking co-occurrence of EH and IIH imaging biomarkers on MRI in a sizable cohort of patients with audiovestibular symptoms. The high prevalence of EH, especially vestibular grade 2, alongside frequent IIH markers such as Meckel’s cave dilatation, empty sella, ON and ONS dilatation, as well as TSS, suggests a potential pathogenetic interplay rather than a coincidental association.

The frequent co-occurrence of multiple IIH markers (≥2) in grade 2 EH patients versus rare in no-EH cases (*χ*^2^ = 12.4, *p* = 0.002) further supports a pathophysiological link between ICP dysregulation and inner ear fluid homeostasis.

*Mechanistic interpretation of TSS-EH negative correlation*: Univariable analyses revealed significant negative associations between venous sinus abnormalities and EH severity. Intrinsic bilateral TSS showed a negative correlation with EH severity (*ρ* = −0.228, *p* = 0.017; OR = 0.262, *p* = 0.013), contrasting with positive associations for ONS dilation (*p* = 0.0368) and slit-like ventricles (*χ*^2^ = 15.375, *p* = 0.0023). This inverse relationship suggests that TSS may impair venous drainage, thereby reducing upstream intracranial ICP transmission to the inner ear via the endolymphatic sac and vestibular aqueduct. Unlike direct CSF pressure effects from ONS dilation or ventricular compression—where elevated ICP more readily disrupts inner ear fluid homeostasis—TSS appears to exert a protective effect against severe EH through altered venous outflow dynamics.

*Clinical symptom overlap and implications*: The predominance of vertigo (69.4%), sensorineural hearing loss (70.4%), and tinnitus (44.4%) in this cohort mirrors IIH symptom profiles. A cohort study reported comparable high rates of vertigo (69.4%), hearing loss (70.4%), and tinnitus (44.4%), with chronic or fluctuating symptoms strongly linked to reduced quality of life and depressive symptoms across various vestibular diagnoses (41). The potential additive burden of concurrent IIH-related pathophysiology, as evidenced by our imaging findings, warrants careful clinical assessment of psychological well-being alongside neuro-otological evaluation in these patients.

Our findings are consistent with prior literature documenting EH in patients with IIH and related ICP disorders. Several reviews and case reports have described EH in patients with SIH showing that both pressure elevations and reductions can alter inner ear fluid homeostasis (2, 42–46). Redon et al. (14) expanded on the neurological perspective, emphasizing that EH may occur secondarily to CSF pressure disorders and should not be regarded as limited to primary otological diseases.

Both our study and the work by Ranieri et al. (46) provide convergent evidence that disturbances in ICP play a pivotal role in the pathophysiology of EH and audiovestibular symptoms. Our study confirms the frequent clinical overlap of symptoms such as tinnitus, hearing loss, vertigo, and aural fullness among patients with either raised ICP or inner ear fluid imbalance, suggesting a shared underlying mechanism. Furthermore, in line with Ranieri et al. (46) our study underscores that effective management of ICP can yield measurable improvements in both neurological and otologic complaints, supporting an integrated diagnostic and therapeutic approach for the affected patient population.

On the other hand, Ranieri et al. (46), reported a much higher prevalence of TSS (96.7%) compared to our cohort (26.9%). This is primarily due to differences in study populations and inclusion criteria. Riggeal et al. (38) studied a consecutive group of patients with a confirmed diagnosis of IIH or IIH without papilledema, conditions that are known to be strongly associated with TSS, as this venous abnormality is present in the vast majority of IIH patients (TSS rates are above 90% in IIH cohorts). In contrast, our study included a broader group of patients presenting for audiovestibular MRI and diagnosed with EH, regardless of clinical suspicion of IIH or elevated ICP. Thus, our cohort reflected a population where TSS is less prevalent and more representative of the general population with inner ear symptoms, rather than a selected IIH population.

The findings of our quantitative MRI-based study of EH and IIH closely align with the case–control study by Tawfik et al. (36), who similarly reported a high prevalence of IIH markers in MD patients, particularly among those requiring surgical management. Both studies confirm frequent coexistence of MRI markers of IIH, including ONS dilation and empty/partial sella, in patients with EH or clinical MD. The studies found a similar prevalence for empty sella (50.9% versus 0%) and ONS alterations (49.1% ONS dilation/57.4% ON tortuosity versus 52% ON changes), supporting the hypothesis of a pathophysiological link between altered ICP dynamics and inner ear fluid imbalance (36). However, our study expands on previous work by applying dedicated hydrops imaging protocols and volumetric analysis of the ON in a broad cohort defined by direct MRI evidence of EH, rather than restricting inclusion to patients with a clinical MD diagnosis. Moreover, our study analyzed a higher number of MRI biomarkers of IIH (additional markers analyzed were: Meckel’s cave dilatation, ON head enhancement, ventricular abnormalities, and venous outflow, as well as scalp and nuchal subcutaneous fat). Our findings highlight a high prevalence of alterations in arachnoid outpouchings, in the ON and ONS, and ancillary findings in EH patients. Furthermore, we found a statistically significant association between vestibular EH severity and ONS alterations, ventricular abnormalities, and increased scalp and nuchal subcutaneous fat, as well as a negative relationship with TSS, underscoring the complex interplay of CSF and venous factors in EH pathogenesis. Overall, our study reinforces the need for integrated MRI protocols that screen for ICP markers in patients with audiovestibular symptoms. Tailored MRI protocols and clinical assessment are urgently required to further clarify overlapping mechanisms that impact on therapeutic management of patients with concurrent EH and IIH features.

The pathophysiological mechanisms underlying the overlap of EH and IIH imaging biomarkers remain incompletely understood. One hypothesis is that raised ICP in patients with IIH could disrupt the homeostasis of inner ear fluid compartments, promoting EH (46, 47). The co-occurrence of Meckel’s cave dilatation and ONS expansion in IIH may reflect broader alterations in cranial CSF pressures, potentially impacting the endolymphatic sac via anatomical channels such as the vestibular aqueduct or through venous outflow alterations. Conversely, underlying microvascular or venous anomalies, which are frequent in IIH and also observed in otological disease, might simultaneously contribute to both inner ear and intracranial pathology (2).

The predominance of symptoms such as vertigo, hearing loss, and tinnitus in this cohort points to a clinical significance of these MRI findings. Although EH has traditionally been linked to MD, the high frequency of its detection in patients with IIH features merits a reassessment of its nosological boundaries and diagnostic implications.

The association between IIH and EH also has potential therapeutic implications. If raised ICP contributes to EH development, then targeted management of IIH might offer symptomatic relief in selected patients experiencing audiovestibular disturbances. Longitudinal studies, including prospective intervention trials and fluid dynamics modeling, are required to clarify causality and impact.

No statistically significant differences in cochlear or vestibular nerve volumes were found between EH grades in this cohort. This suggests that nerve volumetry, as assessed by MRI, may not serve as a robust biomarker for determining EH severity or symptomatic ear involvement in this patient population. A recent MRI study indicated that cochlear nerve caliber may be reduced in cases of severe EH (48), contrasting with our findings, which showed no significant differences in nerve volumes by EH grade or between affected and unaffected ears. This underscores the pathophysiological relevance of our findings in advancing the understanding of intracranial–inner ear fluid interactions. Further research is needed to determine whether nerve morphometry can serve as a robust biomarker across diverse clinical phenotypes.

### 4.1 Limitations

Some limitations of this study are acknowledged. The retrospective design limits causal inferences and exposes the study to potential selection bias, as patients were selected based on audiovestibular MRI indications. Its cross-sectional nature further precludes temporal analysis of the evolution of EH or IIH features. The absence of an age- and sex-matched control group (e.g., patients undergoing brain MRI for headache without audiovestibular symptoms) limits prevalence claims to published norms; prospective studies with matched controls are recommended. Furthermore, the lack of direct ICP measurements, clinical grading of IIH, and detailed vestibular/audiological functional tests limit clinical correlations.

To mitigate these limitations, we included a relatively large patient cohort, which improves the robustness and generalizability of the prevalence findings and allows for a more representative characterization of the association between EH and IIH imaging markers. The large size of our cohort also reduces random variability and provides a stronger foundation for detecting meaningful patterns despite the inherent constraints of retrospective imaging studies.

Future studies should incorporate a prospective design with multimodal assessments, including lumbar puncture pressures, longitudinal symptom tracking, and functional testing to validate and extend these findings.

## 5 Conclusion

In conclusion, this study reveals a substantial overlap between MRI biomarkers of EH and IIH in patients with audiovestibular symptoms, supporting shared or interacting pathophysiological mechanisms. The consistent coexistence of vestibular hydrops and intracranial hypertension biomarkers suggests that disturbances of ICP and cerebrospinal–venous dynamics may influence inner ear fluid regulation. These findings support an integrated approach to neuro-otological assessment, in which evaluation of ICP markers complements inner ear imaging. Radiological protocols should include assessment of structures such as the dural venous sinuses and orbits to detect relevant intracranial features. Prospective studies integrating imaging, physiological pressure measurements, and clinical outcomes are warranted to clarify causality and guide individualized management strategies targeting pressure modulation in selected patients.

## Data Availability

The raw data supporting the conclusions of this article will be made available by the authors, without undue reservation.
